# Unraveling the Evolutionary Diet Mismatch and Its Contribution to the Deterioration of Body Composition

**DOI:** 10.3390/metabo14070379

**Published:** 2024-07-07

**Authors:** Sandi Assaf, Jason Park, Naveed Chowdhry, Meghasree Ganapuram, Shelbin Mattathil, Rami Alakeel, Owen J. Kelly

**Affiliations:** College of Osteopathic Medicine, Sam Houston State University, Conroe, TX 77304, USA; ska029@shsu.edu (S.A.); jjp064@shsu.edu (J.P.); nac069@shsu.edu (N.C.); mxg225@shsu.edu (M.G.); sjm090@shsu.edu (S.M.); rxa133@shsu.edu (R.A.)

**Keywords:** metabolome, ultra-processed foods, dark matter of nutrition, bone, muscle, fat, adiposity, osteosarcopenic adiposity

## Abstract

Over the millennia, patterns of food consumption have changed; however, foods were always whole foods. Ultra-processed foods (UPFs) have been a very recent development and have become the primary food source for many people. The purpose of this review is to propose the hypothesis that, forsaking the evolutionary dietary environment, and its complex milieu of compounds resulting in an extensive metabolome, contributes to chronic disease in modern humans. This evolutionary metabolome may have contributed to the success of early hominins. This hypothesis is based on the following assumptions: (1) whole foods promote health, (2) essential nutrients cannot explain all the benefits of whole foods, (3) UPFs are much lower in phytonutrients and other compounds compared to whole foods, and (4) evolutionary diets contributed to a more diverse metabolome. Evidence will be presented to support this hypothesis. Nutrition is a matter of systems biology, and investigating the evolutionary metabolome, as compared to the metabolome of modern humans, will help elucidate the hidden connections between diet and health. The effect of the diet on the metabolome may also help shape future dietary guidelines, and help define healthy foods.

## 1. Introduction

Over the millennia, patterns of food consumption have changed greatly. For this review, we will use the term ‘ancestral diet’, as the diet changes over evolutionary time scales (‘traditional diets’ are more about the long-standing dietary practices of specific cultures or geographical regions. These diets vary widely around the world and can change over time within a culture). However, the greatest change may be due to the industrialization of food, which has reduced the number of phytochemicals, diminished the metabolome, and introduced chemicals that never previously existed in the food chain. Ancestral humans consumed whole foods, which were minimally processed for millennia. Bipedal humanoid species were present on Earth for at least two million years [1] and the earliest diets consisted of raw fruits. *Homo sapiens* are approximately 300,000 years old [2] and evolved on a diet without processed foods and without farming. What are considered modern humans (Cro-Magnon humans), appeared approximately 40,000 years ago [3]. Agriculture is thought to have begun after the last ice age [4,5], which makes it relatively new, on an evolutionary scale, and humans may still be adapting to it. However, today’s industrialized production of food, estimated to have started in the 20th century [6], has introduced a change to human diets and health that may be several orders of magnitude above the dietary differences between hunter-gatherers and early agricultural communities. Modern industrialized foods, which include ultra-processed foods (UPFs), present a complex paradox. On the one hand, they provide people with the essential nutrients (the three macronutrients, thirteen vitamins, choline, and approximately sixteen major and trace minerals) that are recommended in dietary guidelines, yet they do not mimic whole foods. Conversely, compared to whole foods, industrialized foods lack the complex milieu of phytonutrients, some of which are known to have health benefits [7]. Given the current rise in chronic diseases such as obesity and type 2 diabetes, which began in the 1980s, and the concurrent rise in the ratio of processed foods to whole foods [8], this suggests that essential nutrients may not be playing a key role in preventing chronic disease. For the purpose of this review, the term “essential nutrients” refers to the standard set of macronutrients and micronutrients, while the term “nutrients” will refer to the entire collection of compounds within a whole food.

The evolution of diet is well documented, and a detailed analysis of the literature is beyond the scope of this review. The intention is to highlight some key points in the literature; the following reviews are recommended reading [9,10,11,12]. Nature provided the foods for millions of years and only in recent times has diet changed so radically (e.g., [13]). As described by Alt et al. [11] in their comprehensive review, ‘nutrient cycles’ are part of evolution; nutrients are consumed, metabolized, and excreted by all organisms in an endless cycle, which is required for the survival of future generations. Stable isotope analysis has allowed for insights into ancestral diets, as hominins moved from plant-based diets to being omnivores [12]. For millions of years, hunter-gatherers consumed the wide variety of whole foods they found; however, it was the impact on health that defines contemporary diets, known as “epidemiological transitions” [11]. The direct hallmark of the monumental shift in diet after the agricultural revolution was the rise in dental caries [11,12]. Indirect health effects included a weaker immune system due to the lower micronutrient intakes in a diet composed primarily of carbohydrates [11]. The dramatic decline in the magnitude and diversity of the evolutionary metabolome may also have contributed to the new health paradigm of Neolithic people, and consequently modern humans. Due to the shift from hunter-gatherer to farming practices, people and animals were living in close quarters, resulting in a rise in infectious disease [11,12], and there is some evidence of osteoarthritis from repetitive activities, such as digging soil and chopping trees [11].

One key aspect in the evolution of diet was the consumption of seafood. This provided a higher intake of the long-chain polyunsaturated fatty acids, eicosapentaeonic acid (EPA), and docosahexaenoic acid (DHA). This may have accelerated brain and immune system development [14]. Others argue that terrestrial sources of DHA and EPA were sufficient, and there was an increase in fatty acid desaturase activity approximately 85,000 years ago, which increased the capacity to biosynthesize EPA and DHA, for those early humans who were not consuming seafood [15]. This suggests that nutrient availability has shaped evolution; regardless, the modern diet is low in these key nutrients. Based on this evidence, the evolutionary shift to agriculture greatly improved food security; however, ironically, it did not improve nutritional value (in the broadest sense) or health outcomes.

The latest version of the Human Metabolome Database (www.hmdb.ca, accessed on 31 May 2024) estimates that the current human metabolome consists of approximately 248,097 metabolites, including approximately 32,366 food-derived compounds; the most recently published number of metabolites is 220,945 [16]. This indicates that the modern diet accounts for approximately 13% of the human metabolome. While this seems a small percentage, it is the largest exogenous contributor to the metabolome; drugs and their metabolites are the second largest at 1.3%, see Table 1. Consequently, changes in diet probably result in changes in the metabolome. The magnitude and diversity of the evolutionary metabolome, and how it has changed during evolution, are unknown; it may have even helped shape evolution through genetic adaptations over the millennia [9]. Therefore, the purpose of this review is to introduce the theory that modern diets have a diminished the metabolome, compared to the evolutionary metabolome, and that this shrunken metabolome may be contributing to chronic disease in modern humans.

## 2. Whole Foods and Ancestral Diets

The first whole food that humans receive is their mothers’ milk. Human breast milk is a complex food matrix, composed of macronutrients, micronutrients, and biologics. In addition, the composition changes over time, naturally or due to external factors [17,18,19]. There is still much to learn about human breast milk [20,21], which shows that, like all whole foods, there are properties beyond basic/essential nutrition (energy, vitamins, and minerals). Recent dietary guidelines emphasize improved dietary patterns and diet quality, along with the increased consumption of whole foods to help prevent chronic disease. This change was highlighted in the 2020–2025 Dietary Guidelines for Americans. Furthermore, the development of food-based dietary guidelines (FBDGs) now includes considerations for environmental sustainability and addresses sociocultural factors, such as changing dietary trends [22]. Diet quality is measured using several tools, such as the Healthy Eating Index (HEI) [23]. The maximum HEI score is 100, and a score of 80 or above is considered good diet quality. More recent National Health and Nutrition Examination Survey (NHANES) data, for adults aged 30 or above, show the mean HEI score is below 54 for those with and without diabetes, but higher in those who achieved the Recommended Dietary Allowances (RDAs) for protein [24]. Additionally, the mean HEI score decreased as the number of chronic disease risk factors (obesity, hypercholesterolemia, hypertension, and poor glycemic control) increased. The HEI range across the four groups was 55.7 to 51.1, indicating poor diet quality among all participants [25]. It must be noted that the HEI score is predominately based on the intake quantities of food groups (predominately whole foods) consumed, and only single nutrient items, including sodium and added sugars. Therefore, if a good diet quality mainly consists of whole foods, then it is already recommending the reduced intake of UPFs, although it is not explicitly stated. However, one interesting finding from other observational data is that better diet quality may not offset the effects of UPFs [26]. Hypothetically, UPFs could contribute towards the HEI; however, to gain a good HEI score, a variety of whole fruits and vegetables must be consumed as well as other whole foods, such as lean meats. In addition, avoiding foods that are high in refined grains, sodium, added sugars, and saturated fats will help increase the HEI score—consuming UPFs may counteract this [27].

Ancestral diets were composed of available whole foods (see reviews [28,29,30,31,32]). In general, ancestral diets were composed of available whole foods (plant and animal), of which some were prepared for consumption through cooking (minimal processing) [33]. While a thorough review is beyond the scope of this manuscript, key points will be summarized. Briefly, from an evolutionary perspective (spanning at least 6 million years) the first hominins ate fruits (easily accessible, ready to eat) and then transitioned to being omnivores (*Homo habilis*), and then became more carnivorous (*Homo erectus* → *Homo sapiens*) as more habitats were explored, and more importantly, with the discovery of fire and cooking food [9]. However, humans were never strict carnivores; there was always some intake of plant foods [34], and insects may have also been consumed [35]. The consumption of these complex whole foods must have resulted in a metabolome that had a large number of compounds and was incredibly diverse. It is reasonable to assume that the metabolome changed with seasons, geography, and based on what whole foods were available. If modern humans switch to a Paleo type diet, there are benefits over the short term (6 months), including a reduction in fat mass, and a reduction in risk factors for metabolic syndrome, cardiovascular disease, and type 2 diabetes [36].

From a health perspective, ancestral populations consumed cellular tubers, fruits, and leaves, which were high in fiber and had virtually zero industrial refined carbohydrates, although there is some evidence to suggest that some early humans consumed minimally processed grass seeds [33]. It may be the lack of refined carbohydrates of the ancestral diet that explains lower inflammation in GI microbiota [37]. Kitavan Islanders are one of the few remaining populations that follow a low refined carbohydrate ancestral type diet of tubers and fruits. This population displays none of the classic risk factors (adiposity, hypertension, hyperlipidemia) for ischemic heart disease, compared to the Swedish population [38,39]. Apart from the lower intake of refined grains, the Kitavan Islanders also consume whole foods, which means they have a higher intake of phytochemicals. A higher intake of compounds from whole foods (plant and animal) probably increases the diversity of the metabolome, suggesting that the metabolome may be a marker for cardiovascular health. The Inga community, from Colombia, have had changes to their staple foods, which has led to increased perceptions of food insecurity, malnutrition, and ‘sicker children’, as well as a loss of ancestral knowledge and traditions [40]. Furthermore, UPFs are associated with worse outcomes even when consumed in healthier diets [41]. In addition, more evidence comes from the changes in the dietary habits of migrants, which lead to higher risks of diabetes and cardiovascular disease (changing from whole food diets to industrialized foods) [42,43].

A discussion, although brief, on ancestral diets cannot be complete without mentioning the Paleo diet. Eaton and Konner, in 1985, popularized the idea that shifting from our evolutionary past was a key contributor to chronic disease [44], and published a book entitled *The Paleolithic Prescription: A Program of Diet & Exercise and a Design for Living* in 1989 [45]. A key part of the Paleo concept was the low intake of refined carbohydrates. Loren Cordain published *The Paleo Diet: Lose Weight and Get Healthy by Eating the Food You Were Designed to Eat* in 2002 [46], which was revised in 2010 [47]. More recently, Konner and Boyd Eaton have addressed challenges to the hunter-gatherer model [31], and show that overall, much of the evidence is supportive of “evolutionary health promotion” [48], i.e., modern humans are ignoring their evolutionary past, resulting in chronic disease. Agoulnik et al. [49] and Bland [50], both provide a history of the prior work that led to the Paleo diet phenomenon, especially *The Stone Age Diet: Based on In-depth Studies of Human Ecology and the Diet of Man* by Walter Voegtlin, from 1975 [51].

The Paleo diet is not without its critics, e.g., [52,53]; however, the Paleo diet is focused on consuming whole foods, as are dietary guidelines [22], to achieve weight loss and prevent chronic disease. With this in mind, the UPF debate may be reinforcing the need to move away from the current food trajectory. UPF production has increased steadily since 1990, generating over USD 1.0 trillion in revenue in 2019 [54]. Observational data support the premise that UPFs are contributing to poor health (e.g., [41,55,56,57]). It is very important to note that many UPFs provide essential nutrients, either through mandatory fortification or to supply certain specific nutrients that may be low in diets [58,59]. The genesis of the NOVA classification for processed foods [60] and NOVA itself [61] were devoid of political and commercial influence; they provided a refreshing message with no overt agenda. NOVA is not perfect, just like the dietary guidelines are not perfect. However, NOVA has exceeded its literal definition and has resulted in a *superNOVA* in the world of nutrition. The totality of evidence, although observational, supports the reduction of UPFs. Although some argue that clinical trials are needed [58,59], many dietary guidelines are also not based on empirical proof, such as the five-a-day fruit and vegetable recommendation. Taking a logical and systematic approach, the study by Menichetti et al. [62], which used machine learning (FoodProX), found that while the NOVA system of classification is not perfect, UPFs identified by the algorithm were associated with a higher risk of obesity, diabetes, and hypertension, and reduced serum vitamin status, despite fortification. One clinical crossover study did show that higher UPF consumption, coinciding with a higher intake of refined carbohydrates, resulted in weight gain, compared to an unprocessed diet [63]. This demonstrates that essential nutrients may not be beneficial in preventing chronic disease, especially obesity, and suggests that UPFs are one step further from our evolutionary past and are not contributing to “evolutionary health promotion”.

## 3. The Dark Matter of Nutrition and the Evolutionary Metabolome

**Figure 1:**
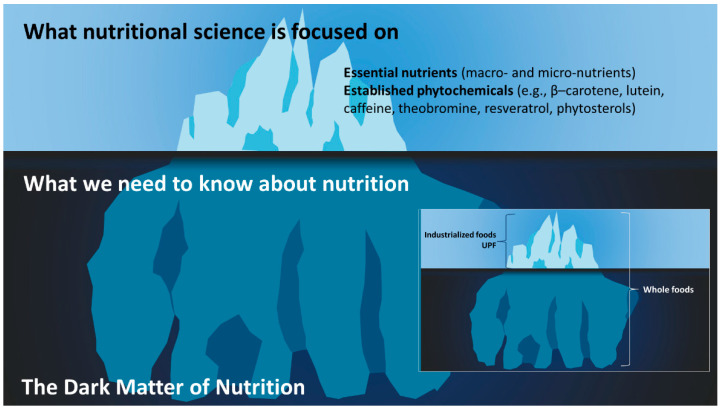
The dark matter of nutrition. The search for the next nutraceuticals is focused on the dark matter of nutrition, showing it is already associated with health benefits. Although dietary patterns/quality endorse whole foods, which encompass the entire iceberg, current dietary guidelines continue to focus on the visible part of the iceberg. In addition, foods are formulated based on the essential nutrients (tip of the iceberg), and this is why UPFs are perceived as healthy [58]. Throughout evolution, whole foods provided a complex and diverse myriad of compounds which contributed to the evolutionary metabolome. While the supplement industry does look below the surface, the image insert shows that the key difference between industrialized foods and whole foods is the inclusion of the entire iceberg.

The biochemistry of essential nutrients is well established; it is in school textbooks, structure–function claims on food labels, and most people have a basic understanding. Essential nutrients consist of proteins (twenty amino acids), fats (nineteen fatty acids reported by What We Eat In America [64]), carbohydrates (composed of monosaccharides, disaccharides, oligosaccharides, polysaccharides, and fibers; it is difficult to estimate the exact number of carbohydrates present in the food chain), water, thirteen vitamins (A, C, D, E, K, thiamin, riboflavin, niacin, pantothenic acid, pyridoxine (B_6_), biotin, folate, cobalamin (B_12_)), choline, and approximately sixteen minerals (Ca, P, Mg, Na, K, Cl, S, Fe, Zn, Cu, Se, Cr, I, Mn, Mo and F). While phytonutrients are not essential nutrients, as they have no established deficiency disease, because some are well known, these can be included in the list, which includes, but is not limited to, β–carotene, lutein, and caffeine. Nutritional science and dietary guidelines have been engaged with these essential nutrients and their Dietary Reference Intakes (DRIs) (composed of Acceptable Intakes, Recommended Dietary Allowance (RDA), Tolerable Upper Intake Levels, Chronic Disease Risk Reduction Intakes, and the Acceptable Macronutrient Distribution Ranges) for decades, which is related to their historical role in deficiency diseases [65]. However, the essential nutrients represent a very small fraction of the compounds in whole foods, the rest being referred to as “the dark matter of nutrition” or “nutritional dark matter” [66] (see Figure 1). Barabási et al. [67] also used the phrase “the dark matter of nutrition” in 2019; however, Bland published their work in April and Barabási et al. published their work in December of that year. This nutritional dark matter is what separates ancestral foods from contemporary industrialized foods (including UPFs), and it is what contributed to the diversity of the evolutionary metabolome.

Nutritional dark matter can be potentially beneficial or detrimental, depending on the situation. Garlic has approximately 2306 known compounds; only 37 are used in health messages. Garlic contains allicin, which can lower trimethylamine-N-oxide formation, a contributor to atherosclerosis, in the gut. Red wine and olive oil contain 3,3-dimethylbutan-1-ol, which also suppress trimethylamine-N-oxide formation [67]. Conversely, Bland [66] proposes that beta-methylamino-L-alanine, from the roots and seeds of cycad plants, contributes to Lytico-bodig disease. Other examples are prions and microRNAs from plants, which can regulate gene expression. These compounds or metabolites in whole foods with negative effects are sometimes referred to as “anti-nutrients”. The more common anti-nutrients include lectins, oxalate, phytates, tannins, phytoestrogens, and goitrogens. As recently reviewed by Petroski and Minich [68], in the context of a varied, whole food diet, there may not be an overall negative effect at physiological levels of these common anti-nutrients. A key point emphasized by Petroski and Minich was that many of the anti-nutrient studies were performed with purified compounds, and at unphysiological levels. In addition, at physiological levels of whole foods, other dark matter compounds, or their metabolites, may counteract any potential negative effects of anti-nutrients. Based on the evidence required to recommend a shift to a plant-based diet [69], as well as the evidence behind food-based dietary guidelines [22], for better health and a reduction in the risk for chronic disease, physiological levels of anti-nutrients seem to have no overt negative effects. Phytonutrients, from the dark matter of nutrition, have also become drugs. An extract of Goat’s rue in medieval times, originally an herbal medicine, to relieve polyuria, became metformin [70]. Other drugs include aspirin, quinine (malaria), and opioids, and plants are still being searched for new drugs [71]. Nutrient synergy is another important point to consider within the dark matter of nutrition. Nutrient synergy is the principle that nutrients have better outcomes when they work together [72]. Simple examples include apigenin and hesperidin, which work together to relieve joint pain [73], or calcium and vitamin D for bone health [72]. Although there is evidence to show combinations of compounds can also be antagonistic [74], there is a large knowledge gap in relation to synergies with the hundreds or thousands of compounds that make up the dark matter of nutrition.

The dark matter of nutrition has been largely ignored, as dietary guidelines focus on the essential nutrients; however, it is being actively searched for new phytonutrients, functional foods, and drugs. Therefore, when consuming whole foods, more phytonutrients are ingested, resulting in a more complex milieu of compounds in the metabolome.

The contemporary human metabolome, with a current estimate of 248,097 compounds by the Human Metabolome Database (www.hmdb.ca, accessed on 31 May 2024), contains food compounds, referred to as the “foodome” by Barabási et al. [67], in addition to endogenous compounds (probably created from raw materials from food) and compounds from the intestinal microbiota (also probably created from raw materials from food), medications/drugs (including metabolites of medications), cosmetics, and environmental toxins or pollutants. Another layer of complexity is that the metabolome differs depending on the location in the human body; the Human Metabolome Database has various samples including blood, saliva, urine, cerebrospinal fluid, feces, and sweat. The gut microflora probably plays a large role in the number and type of metabolites present in the metabolome; this will be discussed later. It may be true that different tissues, such as bone, muscle, and adipose tissues have unique metabolomes; however, these may be helpful in determining the health of the tissues, and future biomarkers of health. It is safe to assume that the evolutionary metabolome was different from the metabolome of modern humans. Discovering the metabolome has been made possible by advancements in liquid chromatography and mass spectrometry (LC/MS). The complexity of the metabolome has also challenged the belief that cellular biochemistry was already well described [75]. At least 220,945 small molecule metabolites have been successfully identified in the human body, with this figure steadily rising each year [16]. Currently, LC/MS has the capability to provide about 10,000 signals from a single sample, making it a key technique for assessing metabolites [76]; however, more refinements will increase the number of signals. These techniques, and others, will be vital to elucidate the role of metabolites in health and disease.

Targeted and untargeted metabolomics are valuable methods for identifying clusters of metabolites that share similar inherent chemical features. Therefore, these approaches are advantageous for finding the metabolites that are linked to different diseases and situations, and subsequently, these metabolites are utilized as biomarkers to track the progression of the disease. For example, there is an association between branched chain amino acids (BCAAs) and diabetes mellitus, obesity, and ischemic heart disease [77]. Handakas et al. utilized nuclear magnetic resonance (NMR) spectroscopy to analyze metabolites from children aged 7–17 years [76] and showed that specific profiles are associated with UPF consumption and the risk of obesity. A prospective study, employing an untargeted metabolomics approach and data from the Atherosclerosis Risk in Communities Study (*n* = 3751), identified twelve metabolites that were associated with UPF consumption and of those, mannose, glucose, and N2, N2-dimethylguanosine were associated with incident chronic kidney disease (CKD) [78].

**Figure 2:**
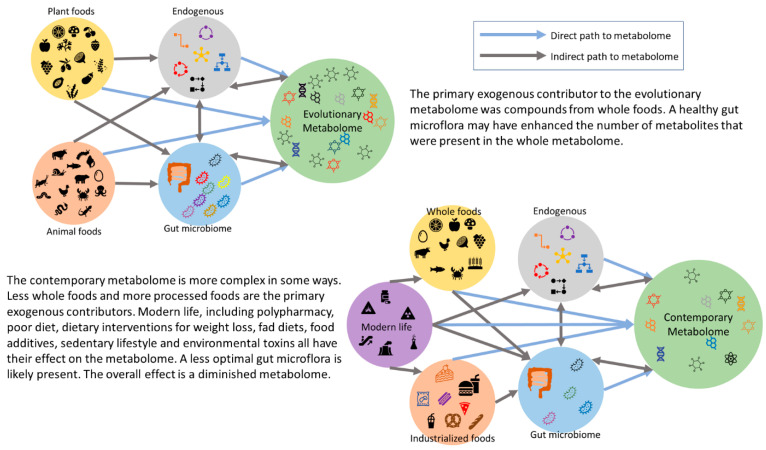
The proposed evolutionary metabolome compared to the contemporary metabolome.

Metabolomics has effectively expanded the complexity of nutritional science and offers a challenge for future dietary guidelines. As a part of adopting this holistic point of view, utilizing databases such as FooDB (https://www.foodb.ca/, accessed on 24 June 2024), Phenol-Explorer (http://phenol-explorer.eu/, accessed on 24 June 2024), and the Periodic Table of Food Initiative (https://foodperiodictable.org/explore/, accessed on 24 June 2024), in nutrition research, offer a more robust understanding of nutrition in health, beyond the essential nutrients. There are several robust studies to show that whole foods have health benefits beyond the essential nutrients [62,79,80,81]. Westerman et al. have explored the nutritional dark matter for compounds with peroxisome proliferator-activated receptor gamma agonist activity, using a tool they developed called PhyteByte [82]. PhyteByte used FooDB and showed that different foods have unique compounds with the same biological function. This has implications for dietary guidelines and health because it allows for more dietary choices, and certain sources are not singled out as a new “superfood”. In relation to our hominin ancestors, this suggests that singular foods were not needed to have a particular desired benefit. Each season or geographic location may have had a food source that conveyed a certain health outcome. The evolutionary metabolome is a theory, following “evolutionary health promotion” [48]. It is difficult to estimate the size and diversity of the evolutionary metabolome (mirroring the rich and intricate metabolome humans evolved with); there is no archaeological evidence. However, it is safe to assume that if one is consuming whole foods, then more compounds are entering the body, resulting in more compounds in the metabolome, in addition to the health benefits. Conversely, consuming a diet that is high in UPFs and low in whole foods would greatly reduce the number of compounds that humans are exposed to; this shrinks the metabolome and results in the contemporary human metabolome (see Figure 2).

Dietary guidelines are promoting better dietary quality to improve overall health, which is reliant on increasing the intake of whole foods. Consequently, a higher diet quality would increase the number of compounds which contribute to the human metabolome. One primary argument to maintain the current consumption level of UPFs is that they provide the essential nutrients to fill potential deficiency/insufficiency gaps in the diet [58,83]. What makes whole foods uniquely different from UPFs is the presence of the dark matter of nutrition, which may have profound effects on human health.

While evidence is needed to support the theory, whole foods, containing the essential nutrients and the dark matter of nutrition, contribute to a metabolome that is larger in magnitude and diversity (similar to the evolutionary metabolome), and the associated health benefits. Conversely, a modern Western diet would have a metabolome that is smaller in magnitude and diversity, and has the associated risks of chronic disease. Metabolomics and network analysis will play vital roles in gaining a better understanding of the role of the dark matter of nutrition in the metabolome, and the subsequent health benefits. All life evolved while consuming whole foods: it is what humans are designed for—would humanity have been as evolutionary successful on UPFs?

### 3.1. Industrialization of Food and the Metabolome

Cattle feeding strategies have changed in the past century to accommodate increasing populations and beef producers are relying more heavily on grain, which may ultimately limit the metabolome. A study by Gómez et al. revealed that different finishing systems and growth rates significantly impact the meat metabolome, with pasture-fed cattle showing higher levels of ATP and fumarate, whereas feedlot cattle had higher levels of succinate, leucine, adenosine monophosphate, glutamate, carnosine, inosine, methionine, glucose 1-phosphate, and choline, highlighting changes in energy, protein, and lipid metabolism [84]. The closest approximation to ancient meat sources is wild game. A nutritional analysis in Europe shows that game meats were lower in fat (less than 3 g/100 g for large species and less than 4 g/100 g for small species), lower in energy (90–113 kcal/100 g), higher in omega-3 fatty acids and minerals such as potassium, phosphorus, zinc, and iron, has a favorable polyunsaturated fatty acid–saturated fatty acid ratio, and an optimal *n*-6/*n*-3 ratio, contributing to its nutritional benefits [85]. Grass-fed beef may also approximate ancient meat sources. A comparison of grass- and grain-fed beef showed that grass-fed beef maintains a better fatty acid profile with a lower total fat content, including 2773 mg less total saturated fatty acid per 100 g of meat, less C12:0 to C16:0 cholesterol-raising fatty acids, higher EPA, docosapentaenoic acid, and DHA, and a more favorable *n*-6:*n*-3 ratio [86]. The latter is known to provide protective benefits against cardiovascular disease and cancer [87,88]. Current fish farming practices use aquafeed, which may have high levels of polyaromatic hydrocarbons (PAHs), which are known carcinogens and genotoxins, and has decreased levels of vitamins A and D [89]. Furthermore, farm-raised oily fish, such as salmon, have a lower omega-3 content, especially EPA and DHA, and have a higher fat content compared to wild caught fish [90].

Agriculture has also shifted to accommodate for the increased population by ensuring maximal crop productivity while minimizing the number of diseased crops. Furthermore, pesticides seem to reduce the levels of phenols and antioxidants and degrade surface proteins [91]. Other practices in modern industrialized fruit production include injecting saccharine to increase sweetness, dyes to increase visual appeal, and waxes to reduce moisture loss and increase marketability (see review [92]). These may be extreme examples; however, they emphasize the focus on quantity and marketability over nutritional quality. Refined wheat is fortified with folate and other vitamins, because they were lost in processing. However, it is unknown what happens to the host of other dark matter compounds that were in the whole grain [93], and that contribute to the metabolome. The metabolome has been greatly reduced with the onset of food processing on a global scale. However, modern agriculture’s focus on only a few crops may further limit the magnitude and diversity of the metabolome. This lack of diversity and shrinkage, in combination with the stagnation of the metabolome, may be contributing to chronic disease.

In sharp contrast to this, the evolutionary metabolome consisted of compounds derived from meat which was higher in glutamine, carnosine, and antioxidant compounds [84], which also offered a higher nutritional value. It is safe to assume that the ancestral diet was devoid of UPFs and was free of synthetic compounds. Ancestral hominins probably consumed fish because of their proximity, their abundance, and there was no requirement for farming [89]. It is estimated that one wild-caught fish contains the same essential nutrients as four farm-raised fish [94], suggesting a higher potential to increase the number of metabolites in the metabolome. Fat intake and health is more controversial [95]; however, linoleic acid (LA), which was markedly decreased in ancestral diets, has shown dramatic increases in the modern Western diet, with an omega-6 to omega-3 ratio between 14 and 25. The excessive intake of LA is converted to inflammatory oxidized linoleic acid metabolites, which are associated with many chronic diseases such as cardiovascular disease and cancer [96]. Furthermore, the 20-fold elevation in the omega-6–omega-3 ratio has been shown to contribute to obesity via mechanisms that influence adipogenesis, adipose tissue browning, lipid homeostasis, the brain–gut–adipose tissue axis, and systemic inflammation [97]. The ancestral diet was devoid of UPFs and was free of human-made pesticides and other synthetic compounds, potentially having a profound effect on the metabolome.

It is estimated that UPFs contribute 50–60% of the energy intake in the Western diet [56]; therefore, UPF consumption may significantly reduce the size and diversity of the metabolome. If the contemporary metabolome consists of 32,366 (www.hmdb.ca, accessed on 31 May 2024) compounds from food (assuming UPF contribute 50–60%), then replacing UPFs with whole foods has the potential to greatly increase the contribution of food compounds to the metabolome. UPFs exist in a dichotomy between the overconsumption of calories and certain essential nutrients, and the under consumption of phytochemicals. Artificial sweeteners affect the gut microbiota and metabolic health. The administration of aspartame has aggravated metabolic diseases by an increase in gut bacteria, which is associated with metabolic diseases, dysregulated glucose metabolism, enhanced insulin resistance, and the increased risk of type 2 diabetes and obesity [98]. Sucralose resulted in dysbiosis in mouse models, as well as intestinal inflammation, which may even affect newborns if present in the mother’s diet, and insulin resistance [99]. There are also concerns regarding preservatives such as sodium benzoate and nitrates in food consumption, leading to health risks. Sodium benzoate induces cell injury, including adverse effects on mitochondrial function by increasing oxidative stress and enhancing pro-inflammatory responses, which can impact DNA damage to mitochondria and impair cellular energy metabolism to cause various metabolic and neurodegenerative disorders, mutagenic, endocrine-disrupting, fertility-reducing, and allergy-promoting potential, as well as produce carcinogenic benzene in the presence of vitamin C [100]. In an acidic environment, preserved foods, including bacon with high amounts of nitrates, can form carcinogenic nitrosamines, which can also increase oxidative stress and inflammation [101]. Nitrates may also lead to endothelial dysfunction, due to alterations in nitric oxide metabolism [102]; however, nitrates may also be beneficial in certain instances [103]. Tartrazine, a widely used food dye, has been linked to increased hyperactivity and attention deficit disorders in children, hormonal imbalances, metabolic dysregulation, and the early onset of puberty [104,105]. A study on immature female Wistar rats showing significant increases in early vaginal opening, serum estrogen, luteinizing hormone levels, uterine epithelial thickness, and follicle numbers, after exposure, suggests long-term health implications such as reproductive cancers, obesity, and cardiovascular diseases [104,106]. Carrageenan, a common food texturizer used for gelling, thickening, and stabilizing properties, increases intestinal permeability, which may lead to “leaky gut” syndrome. A leaky gut causes local and systemic inflammation, impairs nutrient absorption, disrupts gut microbiota, and promotes the growth of pathogenic bacteria [107].

These data suggest that the metabolome can be altered by common food additives, either by being directly bioavailable, or indirectly by altering metabolism or increasing gut permeability. The food industry primarily focuses on producing shelf-stable products, driven by societal and industrial systems that emphasize convenience foods, health concerns, emotional factors, price, availability, societal and cultural influences, environmental and political considerations, and marketing strategies, often prioritizing convenience and marketability over nutritional quality [108,109]. UPFs almost invariably contain amalgamations of flavorings, colorings, preservatives, texturizers, and fortification with nutrients (both synthetic and ‘natural’). Many of these chemicals have not previously existed in the food chain; thus, their role, if any, in the metabolome is unknown. It is safe to assume that the ancestral diet was devoid of UPFs and was free of synthetic compounds.

### 3.2. Fermented Foods, the Gut, and the Metabolome

The area of fermented foods and gut health is gaining research interest. Several recent excellent reviews highlight the fact that fermented foods have a positive effect on microbiome diversity, gut health, and can reduce the risk of obesity, type 2 diabetes, and mood [110,111,112,113]. However, more studies are needed before dietary recommendations can be made. Fermented foods including meat, fish, dairy, vegetables, soybeans, and fruits are considered healthy. Consuming human-produced fermented foods are thought to be relatively new additions to the human food environment and required some genetic adaptation to increase the ability to metabolize alcohol, which likely began as a pre-digestion strategy to enhance nutrient availability in harsh, terrestrial environments [114]. However, hominoids may have consumed foods that were naturally fermented, or in the process of fermenting. There is some evidence to suggest that fruits in various stages of fermentation were consumed by hominins millions of years ago, and some adaption of alcohol dehydrogenase occurred; it seems that even today, many frugivores prefer fruits to be somewhat fermented, with some alcohol content [115]. Nevertheless, fermented foods—as vehicles of key beneficial bacteria from kefir and kombucha to probiotics like sauerkraut, tempeh, natto, miso, kimchi, and sourdough bread—can contribute nutritional value to humans by delivering pre-, pro- and post-biotics [116]. The benefits of this include the production of lactic acid and short-chain fatty acids, the breakdown of anti-nutrients, and the maintenance of GI health. For instance, natto (from soybean) contains a variety of essential nutrients, including vitamin K2 (menaquinone-7), and phytochemicals such as nattokinase, isoflavones, and gamma-polyglutamic acid [117]. All these food compounds contribute to a more complex, and possibly beneficial, metabolome.

Kefir, a fermented milk drink rich in lactic acid bacteria and yeasts, produces bioactive peptides and metabolites such as short-chain fatty acids (SCFAs), which modulate gut microbiota, enhance immune function, reduce inflammation, and improve lactose digestion [118]. Kimchi, a traditional Korean dish, contains vitamins, organic acids, and bioactive compounds that enhance immune function, antioxidant function, support the production of intestinal IgA, and indirectly modulate thiobarbituric acid-reactive substances (TBARSs), demonstrating its potential benefits in GI health, immune system modulation, and oxidative stress management [119,120]. The Human Microbial Metabolome Database (MiMeDB) (https://mimedb.org, accessed on 31 May 2024) has 27,641 total metabolites, including 14,210 human–microbe co-metabolites and 1524 exogenous metabolites from food and other sources, as well as 626 human diseases [121]. Microbial metabolites are becoming more important in human health as there is crosstalk between humans and microbes via metabolites [122]. Apart from the novel metabolites, short-chain fatty acids (SCFAs), amino acids, and vitamins are also microbial metabolites [123].

The intestinal microbiota influences the human metabolome, referred to as the “dark matter of metabolomics”, due to the vast number of uncharacterized chemical signatures, with only 1.8% of spectra currently annotated to aid in chemical structure identification [124]. However, measuring the exact number of bacterial metabolites within the human metabolome is challenging due to the high level of functional diversity in the gut microbiome, varied individual diets, and the complex process of these metabolites entering circulation and affecting different body sites [125]. Modern diets lack the high microbiota diversity of our ancestors and display much lower levels of specific species compared to other current living hominid species (e.g., Chimpanzee), who cultivate a broader range of bacterial taxa [126]. While the inclusion of fermented foods and plant-based carbohydrates with high microbial diversity has influenced the differences between ancestral and modern diets, changes in agriculture and livestock practices have been key contributors to the variations between modern and evolutionary metabolomes, spotlighting that more complex foods and intestinal microbiota result in a larger and more diverse metabolome. Using a contemporary hunter-gatherer population, the Hadza of Tanzania, Carter et al. [127] performed an ultra-deep sequencing analysis of stool samples gathered over one year (2013–2014). They found seasonal shifts in bacterial species as food sources changed, and better microbial diversity, and a microbiome that may be closer to what existed before industrialization. An earlier analysis showed an increase in bacteria that aids in plant digestion, suggesting more nutrients could be extracted [128]. It is evident that the more complex the foods and intestinal microbiota, the larger and more diverse the metabolome.

**Figure 3:**
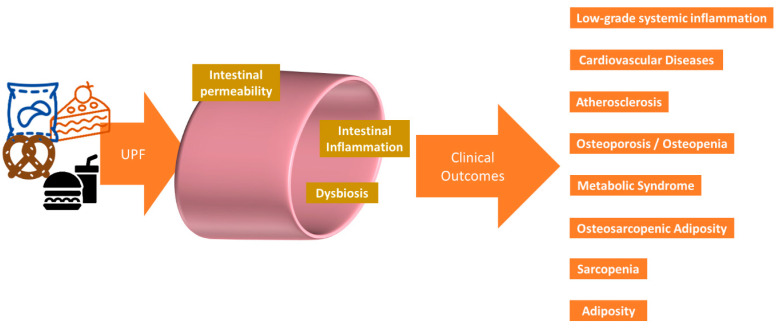
Potential clinical impact of UPF via the gastrointestinal system. UPFs can negatively impact the body by contributing to dysbiosis, low-grade chronic inflammation, bone loss, muscle loss, and adiposity. These result in the clinical manifestations of cardiovascular disease and metabolic syndrome.

Short-chain fatty acids (SCFAs), acetate, propionate, and butyrate are produced from the fermentation of available non-digestible carbohydrates (fibers) by the gut microbiota. These are modifiable by diet, because if absent, then SCFA production will be greatly diminished, and a variety of fibers are needed to produce all the SCFAs [129]. SCFAs have various roles in gut and whole-body health, including maintaining gut integrity and health, selecting for beneficial bacteria, immune system regulation, anti-inflammatory actions, protection against colon cancer, and the prevention of obesity and type 2 diabetes (see reviews [130,131]). Furthermore, in relation to body composition, SCFAs increase postprandial GLP-1 production [131]. Since a good-quality diet would have the potential to produce more SCFAs compared to a poor-quality diet, this suggests that less overconsumption would occur. Modern food production introduced the gut microbes to food additives; these are compounds that were either never present in raw, whole foods, or were present in trace amounts [132]. These compounds and their metabolites probably influence the human metabolome, to some extent, although much more work is needed in this area [133], especially in identifying the bacterial metabolites that contribute to the metabolome [16]. UPFs have introduced the gut microbiota to compounds including nitrites, nitrates, sulfites, polysorbate 80, carboxymethylcellulose, and aspartame. The latter three compounds may increase intestinal inflammation, increase intestinal permeability, and inhibit the growth of beneficial bacteria [134,135,136]. SCFAs are also associated with the development of sarcopenia, possibly via systemic inflammation/metabolic syndrome [137]. UPF additives, particularly emulsifiers and artificial sweeteners, may promote the overgrowth of Collinsella, leading to a pro-inflammatory state and exacerbating conditions such as atherosclerosis and other cardiovascular diseases [138], and possibly osteosarcopenic adiposity (OSA) [139]. A summary of the potential impact of UPFs on GI health is shown in Figure 3. The microbiome has become more important in health research; however, identifying gut microbe-specific metabolites is difficult. The existence of the gut–bone axis [140,141,142], the gut–muscle axis [143,144,145], and the gut–adipocyte axis [146,147,148], shows that fermented foods as well as other modulators of the intestinal microbiota, such as phytonutrients, may be more important in future dietary guidelines.

## 4. Ancestral Diets and Body Composition

The body composition of early humans can only be estimated. There were fundamental changes in body composition during early evolution. As early hominins emerged, the muscle mass of the upper limbs decreased, whereas the lower limbs increased; storing fat (increasing fat mass) also became more important [149]. Weight loss has been at the center of healthcare for decades [150], yet there remains an obesity epidemic. The American Medical Association has recommended the shift away from the clinical use of the Body Mass Index (BMI) to diagnose obesity of the individual in favor of several other measures including body composition analysis [151]. This is a positive step towards returning to the original definition of obesity, which was abnormal or excess fat accumulation that represents a risk to health [152]. The associated health issues are somewhat standardized to the criteria for metabolic syndrome: hypertension, elevated triacylglycerides, lower HDL, and elevated fasting plasma glucose/insulin resistance [153]. This original definition of obesity left room for an individual to have excess fat without a risk to health, which would now be referred to as ‘metabolically healthy obese’; there is some evidence that the number of metabolically healthy obese people is increasing [154]. However, using fat mass to define obesity may result in a rise in the numbers of those with normal weight obesity [155], i.e., healthy body weight with high fat mass; this may offer a new clinical challenge, especially in light of the current trend in GLP-1 agonist drugs for weight loss.

Excess fat mass, especially ectopic fat, and hypertrophic adipocytes, can result in alterations to body composition. Thus, adiposity contributes to bone (osteopenia/osteoporosis) and muscle loss (sarcopenia), eventually resulting in osteosarcopenic adiposity (OSA) [156]. As the clinical definition of obesity changes, there may be a need to redefine other conditions that rely on BMI, such as sarcopenic obesity and osteosarcopenia.

Body composition data from various non-hunter-gatherer, ethnic populations are mixed [149,157,158,159,160,161,162]. There are clear sex differences in body composition; however, altitude [157], ethnicity [158,159,160], and evolutionary changes [149,161,162] can all affect body composition. This implies that body composition is highly responsive to diet and lifestyle, which in turn implies that body composition is a result of the environment. If this is the case, then body composition may also be modified by the metabolome. There is evidence to show that diets that are higher in whole foods are beneficial for bone health [163], muscle health [164], and for preventing adiposity [165]. This may mean that a more diverse metabolome can contribute to the benefits of whole foods in relation to body composition. As the purpose of this manuscript is to introduce the concept of the evolutionary metabolome, a thorough evaluation of the literature on diet and metabolomic profiles, and their effect on body composition, is beyond the scope of this review; however, they will be briefly described.

### 4.1. Focus on Bone

Of the three tissues, including bone, muscle, and adipose tissue, bone is the most likely to survive. Bone size and strength has also changed through evolution [166]. It could be postulated that weaker bones define *Homo sapiens* from our evolutionary ancestors [167]. This adds a higher risk of osteoporosis to the health consequences of abandoning the hunter-gatherer lifestyle. To put the bone loss in perspective, the bone health of bronze age and Neolithic peoples are equivalent to those of modern-day athletes [168]. Osteometabolism is a term used to describe the changes in the metabolism of bone cells in disease states [169], providing a premise that disease is a matter of metabolic dysfunction. Early development work has been performed to identify the metabolomic signatures of postmenopausal osteoporosis, senile osteoporosis and secondary osteoporosis [170,171,172,173,174], with the goal of utilizing them in prevention and treatment strategies. Detecting meaningful changes in bone density requires time, at least twelve months [175]; the necessity of follow-up bone mineral density (BMD) measures is still questioned for those taking antiresorptive treatments [176], showing better bone health measures are required. However, metabolomic signatures may be able to better predict the subtle metabolic changes that lead to future bone loss, or future bone growth.

**Figure 4:**
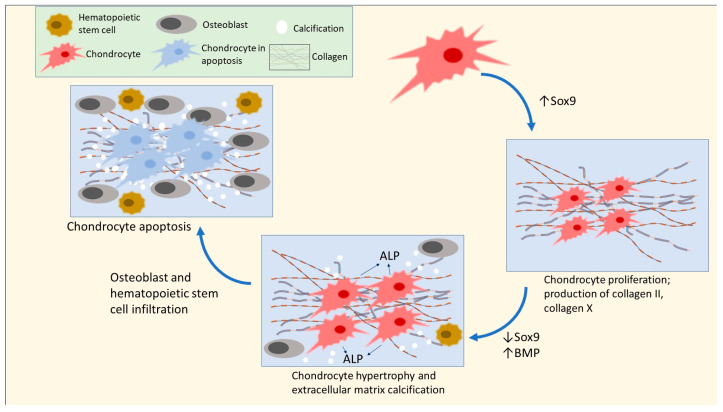
Early stages of endochondral ossification in chondrocytes. This diagram depicts the initial phases of cell division and enlargement in chondrocytes, with a particular focus on the significance of Sox9. Elevated Sox9 expression will promote ongoing cell division of chondrocytes and hinder the initiation of hypertrophy. Reduced Sox9 expression results in the enlargement of chondrocytes and the accumulation of mineral deposits in the extracellular matrix, ultimately causing the death of chondrocytes and the creation of bone. High concentrations of collagen 10 and Sox9 signify an arrest at hypertrophy that stops the production of bones and matrix calcification [177,180,184].

Emerging data indicate that early exposure to UPFs might significantly hinder osteometabolism and increase children’s risk of the development of OSA by impairing the process of the formation of long bones, or endochondral ossification. The early stages of endochondral ossification consist of small chondrocytes, or cartilage-producing cells, in long bones that produce hyaline cartilage. These cells undergo hypertrophy and secrete alkaline phosphatase (ALP), which cause the chondrocytes to undergo apoptosis. Subsequently, osteoclasts will break down the matrix, enabling the infiltration of osteoblasts and blood vessels to form bone [177,178,179,180] (see Figure 4). Zaretsky et al. [181] fed young female rats UPFs (including a roll, a hamburger, and French fries), together with a high-calorie soft drink, compared to a control standard rat chow, for a duration of six weeks. The UPF diet resulted in substantial declines in several parameters related to endochondral ossification, including a decrease in the length of the femur, weakened outer layers of the bones (cortices), and the presence of lesions and enlargement in the growth plates of the tibia. In addition, the long bones of the UPF-fed rats showed reduced mineralization and the occurrence of avascular non-mineralized cartilage lesions. At the molecular level, the mRNA sequencing of the genomes of the rats in the UPF group revealed an upregulation of genes coding for several proteins that sustained chondrogenesis, halted the growth of chondrocytes at the hypertrophy stage, and delayed the process of calcification of the extracellular matrix. The analysis also revealed the presence of inhibitors for proteins that promote the transition from cartilage development to matrix calcification and bone formation, including collagen 2, collagen 10, bone morphogenetic protein (BMP) inhibitors, and the sex-determining region Y (SRY)-box 9 (Sox9). The lower levels of calcium and phosphorus in the UPF diet help explain some of the findings. Similar findings are in humans, where insufficient calcium and phosphorus intakes are present, due to increased UPF consumption, particularly among children [182,183].

Zaretsky et al. [181] were the first researchers to establish a mechanistic connection between the intake of UPFs and the stunted growth of long bones in an animal model by way of elevated Sox9 expression. In their study, the researchers noted increased levels of Sox9 concurrent with the increased expression of downstream genes responsible for continued extracellular matrix growth, such as Gdf10 and Acan. The full implications of these findings on endochondral ossification in humans have not been completely understood. Further studies are needed to determine if similar molecular processes are involved in humans, and if there is a specific risk of developing OSA in children and adolescents due to impaired endochondral ossification from consuming excess UPFs. Rat chow does contain the essential nutrients at levels designed to prevent deficiencies [185]; therefore, the result was no deficiency in bone growth parameters [181]. However, while the UPF diet contained less calcium and phosphorus, which may help to explain some of the results, the authors suspect other components of the UPFs were having an effect. One intriguing observation from this study is that standard rat chow could also be considered an UPF, as it is made from industrialized ingredients. This provides some evidence that having the essential nutrients can help prevent metabolic dysfunction due to deficiencies. Providing just enough nutrients to prevent deficiency—the minimum—will not help to identify the optimal metabolic conditions for bone health. The UPF diet, used by Zaretsky et al. [181], may also contain more additives compared to the standard chow, which may affect the outcome. Another possibility is that consuming UPFs necessitates a higher intake of calcium, i.e., an incorrect calcium–phosphorus ratio, due to the higher phosphorus content (phosphate additives) of UPFs. Regardless, this is a stimulating question, which may be explained using metabolomics.

The systematic review on dietary patterns and bone health, performed as part of the 2020 Dietary Guidelines for Americans, suggests that a more plant-based diet with fish and meat, but limited in processed meats and added sugars, is better for bone health [186]. An earlier scoping review found that the Mediterranean diet, and higher Healthy Eating Index scores, benefited bone health [187]. More recent reviews suggest a healthy balanced diet is best for bone health [188,189]. These data suggest a diet higher in whole foods, and the resultant more diverse metabolome, benefits bone health. Fermented food may also benefit bone health; a prospective cohort study of postmenopausal Japanese women including 1417 subjects equal to, or older than, 45, indicated that there was a significantly reduced risk of osteoporotic fractures in those who habitually consumed the fermented food natto [117].

Interestingly, the risk for fracture seems to be higher for vegans [190,191,192], although some disagree and suggest key essential nutrients to supplement vegan diets [69,193,194]. Vegans are at risk of vitamin B_12_ insufficiency or deficiency. Vitamin B_12_ is vital for energy metabolism, so a deficiency can result in weakness and fatigue (low physical activity), which can contribute to sarcopenia, especially in older populations [195]. Low physical activity in the elderly may also result in adiposity. Moreover, vitamin B_12_ insufficiency or deficiency has been associated with decreased osteoblastogenesis through higher homocysteine levels, leading to lower bone mineral density and an increased risk of osteoporosis [196]. Folate deficiency, or a functional folate deficiency due to a vitamin B_12_ deficiency, can elevate homocysteine, and it has been linked to metabolic syndrome, reduced collagen synthesis, and obesity [197,198]. Folate also plays a role in DNA methylation, epigenetic modification, and the impaired gene regulation of fat synthesis, storage, and breakdown may increase lipid accumulation [198,199]. Furthermore, elevated homocysteine due to a vitamin B_6_ deficiency can suppress the expression of lysyl oxidase and the synthesis of collagen required for the bone matrix and is related to an increased risk of bone fractures [196,197]. However, folate and vitamin B_6_ deficiencies are less likely in vegans. A key question is what constitutes a modern vegan diet; is it high in refined carbohydrates and not including enough of a variety of fruits and vegetables and other plant foods (e.g., herbs)? Sotos-Prieto et al. [200] addressed this and found that a healthy plant-based diet (lower in refined carbohydrates and added sugars) had better bone outcomes compared to those on an unhealthy plant-based diet. Modern plant-based/vegan diets, consisting of prepackaged meals, may have the same effect as UPFs. Vegans seem aware of the potential nutrient insufficiencies and many use supplements; however, iodine may be a nutrient of concern [201]. Overall, a vegan diet, emphasizing fresh whole foods, and low in refined grains, may have benefits due to the enhanced metabolome.

Other reviews have focused on phytonutrients and bone health. Overall, carotenoids, flavonoids, and polyphenols may improve bone health via various mechanisms, especially via antioxidant mechanisms [7,202,203,204]. Taken together, this supports the premise that a diet higher in whole foods and lower in UPFs supports bone health, possibly due to the larger, more diverse metabolome.

### 4.2. Focus on Muscle

Muscle does not survive in fossil records; however, based on the assumption that hunter-gatherers were in energy deficit for the majority of the time, it can be postulated that they were lean, as nutrients would have been transferred back and forth between muscle and other tissues [205]. The consensus is that hunter-gatherers had a small body size, compared to modern humans [206]; the reduction in the size of the fauna during evolution may help explain this—smaller prey cannot support larger body sizes [207]. Regardless, the leanness of hunter-gatherers suggests a lower a fat–muscle ratio, and this may be an important marker. This makes sense given the lifestyle and available energy and nutrients. In addition, a leaner hunter-gatherer would be more suited to activity compared to an obese human or even a power athlete. The systemic metabolic effects of sarcopenia include insulin resistance, dyslipidemia, and hypertension, and possibly a decrease in resting energy expenditure and increased adiposity [208]. At the myocyte level, changes in muscle fiber type, satellite cell deregulation, inflammation, and oxidative damage are present in sarcopenia [209]. A small study investigating the metabolic phenotype of sarcopenia found that levels of very-long-chain fatty acids, dicarboxylic acid carnitines, and citrulline were associated with sarcopenia, pointing to mitochondrial dysfunction [210]. In a similar study, even though the compounds were not the same, the findings suggest that the metabolites affected by sarcopenia may be modifiable by diet [211]. Other studies have small sample sizes with heterogeneous outcomes [210,212,213,214,215]. Overall, these data strongly suggest that the changes in muscle metabolism due to the metabolome requires more investigation.

Overall, a poor-quality diet is associated with sarcopenia, and an anti-inflammatory pattern diet may be the best strategy to prevent sarcopenia [216]. Higher fruit and vegetable intakes (whole foods) may have a beneficial effect to lower the risk of sarcopenia, although the lack of clinical studies is hindering solid recommendations [217]. Vegan diets, although hypothetically containing a more diverse metabolome, are not currently recommended for sarcopenia [218]. The systematic review on dietary patterns and sarcopenia, performed as part of the 2020 Dietary Guidelines for Americans, did not find enough evidence to recommend a particular diet to prevent or treat sarcopenia [186], so more work on dietary patterns (including various intakes of UPF), the metabolome, and sarcopenia is warranted. Phytochemicals have been shown to improve muscle function. The common example is the role of caffeine in improving exercise performance; however, others including quercetin, anthocyanins, and ellagitannins may also improve muscle size and recovery (see reviews [7,219]). Overall, a diet higher in whole foods, with the resulting increase in the diversity and size of the metabolome, probably contributes to muscle health.

### 4.3. Focus on Adipose

Obesity is the primary public health issue globally [220,221], and UPFs may be contributing [55,222] directly and indirectly. A high-quality diet is recommended by the Dietary Guidelines for Americans as a strategy to maintain a healthy body weight, ideally containing more whole foods. Furthermore, a better diet quality is associated with more weight loss in the shorter [223] and longer [224] term, suggesting some connection with whole foods and better energy homeostasis. A key concern regarding excess UPF consumption is weight gain [41,55,225]. Data from Hadza about hunter-gatherers (Northern Tanzania) showed that total daily energy expenditure (kcal/d), as measured by the doubly labeled water method, did not significantly differ from U.S. and European adults (Western), and farmers from Bolivia, Nigeria, and Gambia. This implies that total daily energy expenditure is an evolved trait and largely independent of lifestyle factors [226], placing a higher emphasis on improving body composition parameters. Maintaining lean body mass (bone and muscle) and limiting adiposity throughout life is important for healthy aging, and remaining healthy [227]. At this point, excess weight, or obesity, is considered a global health crisis [228].

Although body fat is engrained to visually assess an individual’s health on physical examination, or by mere encounter, there are more informative ways to assess adiposity. The current clinical diagnosis of obesity relies on BMI, which indirectly estimates body fat in its relation to an individual’s height and weight. However, BMI neglects to account for changes in body composition—hence, a fat–muscle ratio of 4:7 and 1:9 could both indicate obesity as measured with BMI [229]. It is clearer now that fat mass’ negative effects are more related to the location of the adipose tissue, which can be assessed using a body composition analysis [156]. New technologies are needed to better assess more instances of ectopic fat, such as bone adiposity. Adiposity is a risk factor for numerous other chronic diseases, including type 2 diabetes, cardiovascular disease, COPD, some cancers, osteoarthritis, and mental health issues [230].

The logical assumption is that early *Homo sapiens* were leaner (less body fat), which is supported using comparisons to modern day hunter-gatherers. Female Hazda hunter-gatherers had 24% body fat while males had 9%, as measured by skinfold, similar to findings in other contemporary hunter-gather populations [231]. The Savanna Pumé hunter-gatherers from Venezuela had at least two-fold less body fat compared to a reference U.S. population, as measured by upper-arm fat area (mm^2^) [232].

In an interesting case study, a participant was calorie restricted before an ultra-endurance event to simulate a hunter-gatherer scenario and compared to a participant who was not calorie restricted [233]. The results highlighted the intense physical stress our ancestors were under, including poorer sleep quality, elevated creatine kinase (muscle breakdown), proteinuria, weight loss, and dehydration. Another case study followed a participant who started a Paleo diet for 12 months, where they collected detailed dietary intake data [234]. The participant’s mass increased by 9.5 kg by the end of the 12 months; however, they only had 10.8% body fat. The diet used correlated well with Paleo diet principles and consisted of 46.2% animal products, 7% vegetables, 7% fruits, 11% nuts and seeds, and 6% dairy (the remaining came from various sources). A noteworthy finding was that the Paleo diet exceeded the Nordic Nutrition Recommendations for micronutrients. While this was data from a case study, its meticulous data gathering provides preliminary information that the Paleo diet, without calorie restriction, may support the accrual of muscle mass while keeping fat mass low. Considering the dietary intake consisted of mostly whole foods with minimal processing, this suggests that the Paleo diet may promote a diverse metabolome.

As recently reviewed, numerous phytonutrients can modulate adipose tissue and have potential as obesity treatments [235]. While many of the phytonutrients, including the various polyphenols, alkaloids, terpenoids, saponins, and anthocyanins, are studied in isolation, there may be synergies, considering the thousands of compounds present in whole foods. With this in mind, Urasaki and Le showed that a blend of nine phytonutrients (berberine, luteolin, resveratrol, fisetin, quercetin, fucoidan, EGCG, hesperidin, and curcumin) reduced weight gain, visceral adiposity, and inflammatory cytokines in a mouse model of obesity [236]. There are preliminary studies to show that there may be metabolomic signatures associated with weight loss [237,238,239]. In addition, there are metabolomic differences between those who are metabolically healthy and metabolically unhealthy obese [240], and certain metabolomic signatures are also associated with visceral fat [241]. The changes to the adipocyte in obesity include hypertrophy, increased inflammatory adipokines, the redistribution of adipose to ectopic sites, and the production of lipid-filled vesicles [242,243]. Evidence suggests that a healthy dietary pattern (high in whole foods) would help to reduce the risk of adiposity [244]. The Mediterranean diet (high in phytonutrients [235]) remains one of the most recommended diets to prevent and treat adiposity [245]. There is a wealth of data related to the metabolomic phenotype of obesity, including the differences between metabolically healthy obese and metabolically unhealthy obese individuals (e.g., [246,247,248,249]); however, reviewing these data is beyond the scope of this review.

Taken together, the data presented show that whole foods, and the resultant richness of phytonutrients and diverse metabolome, are important in bone and muscle health and in reducing adipose tissue accrual. A higher fruit intake reduced the risk of OSA in Korean postmenopausal women [250]. The role of essential nutrients in preventing and treating OSA has already been reviewed [251,252], and a whole food diet can supply these nutrients. This suggests a diverse metabolome may have a role in modulating body composition, ultimately preventing OSA.

## 5. The Ultra-Processed Food Conundrum

*In the year 4545*

*Ain’t gonna need your teeth, won’t need your eyes*

*You won’t find a thing to chew*

*Nobody’s gonna look at you*

(Zager and Evans, 1969) [253]

Zager and Evans released their song, “In the Year 2525 (Exordium & Terminus)” in 1969, from their debut album “2525 (Exordium & Terminus)”. This was released at a time when fewer ultra-processed foods (UPFs) were available. Given the current direction of food production, and to some extent current dietary guidelines, these words seem prophetic; they predicted that future food would be so processed, it would not even require chewing.

Dietary guidelines are tortuously linked with the food chain (including food regulations and health claims). One of the main arguments against NOVA, and reducing UPFs, is that some UPFs contain essential nutrients, which may be low in diets, and therefore are needed to maintain public health [58,59]. Using the presence of essential nutrients to imply that UPFs are ‘healthy’ is somewhat flawed. Dietary Reference Intakes (DRIs) are not an indication of optimal levels of a nutrient; they indicate a healthy individual is consuming enough of a nutrient to prevent a deficiency disease [254]. Remarkably, despite the historical and current focus on DRIs, there has never been a randomized clinical trial to investigate a diet containing all the nutrients at their DRI level, compared to other doses, in any population, for any outcome. Despite this, they are ubiquitously provided as dietary targets for individuals to achieve, both in the community and even for hospitalized patients. More importantly, they are used to measure a food’s healthfulness [58,59]. With food fortification, the essential nutrients are possibly more available than they ever were throughout evolution, yet the incidence and prevalence of chronic diseases and other health issues question their efficacy in preventing them. This leads to the hypothesis that the answer to human health lies beyond the essential nutrients.

Children and adolescents are more likely to consume UPFs; marketing and advertising campaigns target children [255] and UPFs are convenient for packed lunches. Additionally, UPFs are readily available in school environments [256]. Excess UPF consumption in children may potentially impede endochondral ossification [181] and increase weight gain [257,258], resulting in early adiposity and not achieving peak bone mass, ultimately increasing the risk for OSA.

**Figure 5:**
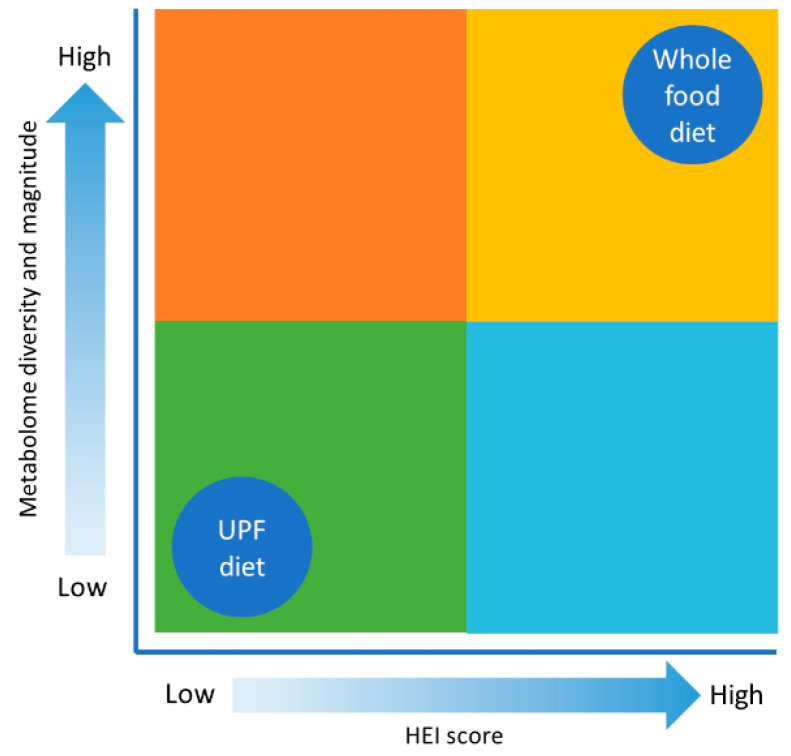
Metabolomic diversity and diet. This figure illustrates how metabolomic diversity and magnitude changes as diet goes from being based on whole foods to a diet consisting of industrialized food. In addition, HEI decreases as the diet is composed of more UPFs.

Recent publications of opinions on UPFs, from experts and expert societies [58,59,83,259,260], highlight the disconnect between dietary guidelines, nutritional science, and healthy eating. Current dietary guidelines remain in a state of flux from the reductionist beliefs of the 20th century, focusing on individual nutrients and their individual effects on the body (preventing deficiencies), to a holistic perception of nutrition, more focused on diet quality/dietary patterns, and whole foods [22,65]. While there is no strong direct evidence to say UPFs are doing harm, there is also no strong evidence to the contrary. Providing the essential nutrients can be interpreted as providing the minimum, as it relates to the number of compounds in whole foods, and the potential benefits of the dark matter of nutrition. Therefore, the contribution of a food to the human metabolome is one such alternative measure of a food’s healthfulness. Processing is a matter of removing the compounds that make whole foods healthy, and in some ways, the supplement industry is filling these gaps. Whole foods are needed to improve diet quality. Figure 5 proposes a means to assess diets, based on their potential to improve diet quality (HEI) and contribute to the metabolome.

### Illustrating How Whole Foods Contribute to the Metabolome

For illustration purposes, two diets were created; one diet was predominately composed of UPFs and the other was predominately composed of whole foods. For the composition of the diets and the number of metabolites in each diet, see Table 2. The summary of the nutrient analysis (Axxya Systems Nutritionist Pro™ Version 8.1.0) and the contribution of whole foods to the metabolome of the diets is shown in Supplemental Table S1. When designing the UPF and whole food diets, there were no energy or macronutrient targets; the main goal was to have one diet contain as much UPFs as possible and the other have as much whole foods as possible (within reason).

The whole food diet contained an estimated 185,937 food compounds while the UPF diet contained 71,717. It is important to note that these preliminary data are not based on the amount of a food—it merely shows the total number of compounds per individual food. The concentration of compounds is expected to have an effect on the metabolome; however, this is not represented. UPFs are not included in FooDB; therefore, the manufacturers websites were checked for the number of ingredients per label claim, if nutrition facts/food labels were available. For example, the diet cola drink has at least six ingredients (the number of compounds that made up the natural flavors were not indicated on the label). To minimize bias, wherever a baked good was included in the diet, flour was used to estimate the metabolites; however, the difference in metabolite number between commercially used refined flour and whole kernels is not known. Many food compounds, including essential nutrients, are lost in processing. Refined wheat is fortified with folate because it is lost in processing [93]; it is unknown which other dark matter compounds are lost. Therefore, there may be some over-estimation of the number of metabolites in the UPF diet.

For both diets, any spices and herbs used during cooking were not included, and neither was the effect of cooking on any of the food compounds/metabolites. These estimates also ignore any post consumption elimination and/or intestinal microbiota transformation or utilization of the food compounds. Meat contributes many compounds; if the contribution of meat is removed from both diets, then the whole food diet would be reduced to 100,955 food compounds. This may provide some estimate of the vegetarian metabolome. Conversely, if more meat was added to the UPF diet, it would increase the number of food compounds. However, the point was to have a diet high in UPFs and fresh meat is not considered a UPF, although chicken nuggets were counted as chicken. This exercise shows that a whole food diet will contribute more food compounds to the human metabolome compared to a diet high in UPFs, since diet is the most significant exogenous source of compounds for the metabolome. While FooDB focuses on whole foods, adding all foods may provide a database to aid in future dietomics (omics technologies to assess the healthfulness of a diet or food) research. In addition, the effects of minimal processing (e.g., cooking) on the food compounds should be considered.

There are several benefits of the whole food diet, e.g., it is higher in fiber, has a better omega-6–omega-3 ratio, better potassium–sodium ratio, and a high choline content. There are also some nutrient intakes which may be considered unhealthy per dietary recommendations, e.g., higher cholesterol, higher phosphorus, and lower vitamin D intake. However, for vitamin D, there are no good food sources (because it can be biosynthesized) and it is usually fortified in processed foods, see Supplemental Table S1).

There is no single entity to blame for UPFs. The food industry follows the dietary guidelines. When fat was ‘bad’, lower fat and fat-free foods were created. Currently, added sugars are ‘bad’, so foods with lower sugars or zero sugars have been developed. While breastmilk is the gold standard for infant formulae (an UPF), whole foods do not seem to be the gold standard for industrialized foods. Over the last half century, the DRIs have offered relatively easy nutritional targets for the food industry. With dietary guidelines slowly shifting to FBDGs (dietary pattern/quality), at some point they should exceed the limitations of DRIs [261]; what will this mean for the food industry? The fundamental shortcomings of the current dietary guidelines as they relate to what constitutes a healthy food must be recognized, and there must be an effort to work together to create unbiased dietary guidelines for the 21st century.

## 6. Discussion

The goal of this review was to introduce the concept of the evolutionary metabolome and spark interest in a potentially new area of research. The concept of this review builds on, and connects, the literature related to human and diet evolution, hunter-gatherer lifestyles, phytonutrients, gut health, body composition, and UPFs. The human metabolome exists, and more data are being added through network analysis studies, such as those by Barabási et al. [62], related to how the metabolome affects health. It is important to note that nutritional science, at its core, is systems biology. The evolution of diet is an area that has been extensively researched. Foods, in Western countries, have shifted to being at least 50% UPFs [56], and away from the whole foods that nature provided throughout evolution. Overall, globally, modern dietary guidelines are recommending better dietary patterns and diet quality, and the consumption of more whole foods [22]. Evidence was discussed that shows high quality diets can benefit health and body composition, especially OSA (the cluster of osteopenia/osteoporosis + sarcopenia + adiposity). Body composition changed throughout hominin evolution, driven by large changes such as walking upright, geological changes (ice age), and more recently by the introduction of agriculture, industrialized food, and sedentary, stressful lifestyles. This provides the first assumption of our concept: whole foods are better for health and body composition.

It is difficult to talk about diet without implicating health; ‘diet’ and ‘health’ seem synonymous. This leads to the question—why are whole food diets better for health? Evidence was presented to show that while the essential nutrients are important to prevent clinical deficiency diseases, phytonutrients may be the key players in modulating health. More recently, these phytonutrients have been referred to as “the dark matter of nutrition” [66]. Therefore, the second assumption of our concept is as follows: the health benefits of food extend beyond the essential nutrients.

The next assumption is as follows: modern foods (UPFs) are low in phytonutrients compared to whole foods. For the most part, many phytonutrients are lost in processing, so UPFs would be low in these compounds. Paradoxically, the search for new food ingredients with health benefits (nutraceuticals) and drugs, is within the dark matter of nutrition (the part of the food which is diminished in processing and is not focused on in dietary guidelines). An example of this was provided, as shown in Table 2, which shows that a UPF diet would probably contain less phytonutrients than a whole food diet (71,717 versus 185,937, respectively). These numbers are based on data obtained from FooDB. These food compounds may be metabolized, by various methods (from enzymes → gut bacteria), to other compounds, which may result in the activation (biological effect) or deactivation (no biological effect) of the metabolite.

This leads to the next assumption: ancestral humans had a high intake of whole foods; therefore, they had a high intake of phytochemicals, and at physiological levels. The physiological level of compounds is important, as it does not mean that high doses would have a greater benefit than what was intended by nature. Furthermore, the absence of overt negative effects from whole foods at physiological levels suggests that “anti-nutrients” present at physiological quantities may not have any net negative effect in the context of a good diet [68].

Finally, the greatest exogenous influencer of the metabolome is food, and this leads to the following hypothesis: moving away from the evolutionary metabolome may contribute to chronic disease in modern humans. The evolutionary metabolome, the metabolome that was present during the evolution of hominins to humans, over several million years, may even be required for optimal health. The change from hunter-gatherer to farming practices may have limited the metabolomic diversity, as the same foods were relied upon year after year. The shift to industrialized food (UPFs) further solidified monoculture practices and may have led to further reductions in metabolome diversity. Whole food diets may mimic the evolutionary metabolome.

How the evolutionary metabolome fits into the future of nutritional science is not certain. However, embracing the dark matter of nutrition will help to truly understand the deep connection between food and health. A question that needs urgent attention is as follows: how do food additives affect the metabolome? In this way, metabolomics will be a crucial tool for understanding the health benefits of whole foods and their role in the prevention and treatment of disease, compared to UPFs. The effect of a food on the metabolome may also help to determine which foods are ‘healthy’.

The evolutionary metabolome may have helped to maintain good body composition. Body composition changed throughout hominin evolution, driven by large changes such as walking upright, geological changes (ice age), and more recently, by the introduction of agriculture, industrialized food, and sedentary, stressful lifestyles. A healthy diet and exercise can modify body composition; however, the anabolic roles of the evolutionary metabolome in bone, muscle, and adipose tissues (OSA) need investigation. It can be argued that physical activity has the greatest effect on body composition; however, the evolutionary metabolome may have had bone and muscle-sparing properties.

All this suggests that the metabolome may have an endocrine effect, affecting all tissues, including bone, muscle, and adipose tissues. Modern chronic diseases may be diet-related because diet is the largest exogenous contributor to the metabolome. More studies that compare the scale of the evolutionary metabolome to the contemporary metabolome of modern humans, and the resultant health outcomes, are needed. Analyzing the relationships between UPFs, OSA and the metabolome may allow for greater insight into the role of diet in healthy aging. There is also a great need to explore the contribution of our diminishing metabolome to other health conditions and diseases. Nature provided the nutrients to help shape humans over millions of years; now, humans are deciding what nutrients they should be having.

The evolutionary metabolome adds to evidence for the view that whole food diets are what is best for humans, and provides a reason to consume more whole foods and increase the consumption of whole plant-based foods. This would then be essentially eating to improve one’s metabolome—the metabolomic diet. While this review may seem critical of UPFs, no single UPF is innately bad; the dose of UPFs in people’s diets is the issue.

## 7. Conclusions

Overall, this review suggests that more is unknown about food and health than is known. The metabolome offers an objective foundation for investigating food and health. There are tools available to handle this level of data and it may be time to apply them to nutrition and to dietary guidelines. The latter could be referred to as the dark matter of the guidelines. It must be remembered that current metabolomic studies are based on modern humans consuming Westernized diets. It is crucial to unravel the role of the mismatch between the evolutionary metabolome (whole food metabolome) and the metabolome of the modern food environment in health.

## Figures and Tables

**Table 1:** Origin of metabolites in the human metabolome from the Human Metabolome Database (www.hmdb.ca), accessed 24 June 2024.

Total *	Exogenous	Endogenous	Food	Plant	Microbial	Toxin or Pollutant	Cosmetic	Drug	Drug Metabolite
248,097	35,830	221,039	32,366	146	172	157	17	2379	909

* Includes all biospecimen types (blood, saliva, urine, cerebrospinal fluid, feces, sweat, breast milk, bile, amniotic fluid, other).

**Table 2:** Estimated number of metabolites from a de-identified ultra-processed food diet compared to a whole food diet.

Foods	Food Compounds (No.)	FooDB ID	FooDB Name
**ULTRA-PROCESSED FOOD DIET (24 h INTAKE)**			
Breakfast			
Coffee, brewed	4433	FOOD00059	Coffee
Sugar coated flakes of corn	80	FOOD00270	Breakfast cereal
Liquid coffee creamer	11	Not listed	
Almond milk, sweetened	80	FOOD00937	Almond milk
Morning snack			
Glazed donut	3969	FOOD00799	Flour
Cola drink	6	Not listed	
Lunch			
Chicken nuggets, 6 pieces	42,460	FOOD00329	Chicken
French fries, large	4470	FOOD00175	Potato
Diet cola drink	8	Not listed	
Afternoon snack			
Protein bar 30 g, chocolate flavor	60	Not listed	
Tap water		N/A	
Dinner			
Pizza, cheese topping, thin crispy crust, frozen, baked	39	FOOD00933	Mozzarella
	4194	FOOD00171	Tomato
	3969	FOOD00799	Flour
Chocolate chip cookies	3969	FOOD00799	Flour
Tap water		N/A	
Evening snack			
Pretzels, hard	3969	FOOD00799	Flour
TOTAL	**71,717**		
**WHOLE FOOD DIET (24 h INTAKE)**			
Breakfast (omelet with spinach, shallots and mushrooms)			
Eggs, fried	98	FOOD00619	Egg
Spinach, cooked, from fresh, fat added in cooking, NS as to type of fat	4227	FOOD00178	Spinach
Shallots	4013	FOOD00243	Shallot
Mushrooms, cooked, from fresh, fat added in cooking, NS as to type of fat	4505	FOOD00547	Common mushroom
Coffee, brewed	4433	FOOD00059	Coffee
Morning snack			
Peanuts, Spanish, raw	4130	FOOD00016	Peanut
Tap water		N/A	
Orange	4333	FOOD00057	Sweet orange
Lunch (grilled chicken breast with mixed vegetables, mixed fruit for desert)			
Chicken breast, grilled, meat and skin	42,460	FOOD00329	Chicken
Pepper, bell or sweet, red	4391	FOOD00880	Red bell pepper
Onions, chopped	4514	FOOD00006	Garden onion
Onions, scallion, or spring green	4262	FOOD00241	Welsh onion
Tomatoes, cherry, fresh	3977	FOOD00172	Cherry tomato
Spinach, cooked, from fresh, fat added in cooking, NS as to type of fat	4227	FOOD00178	Spinach
Oil, olive	8	FOOD00909	Olive oil
Cantaloupe	4010	FOOD00984	Cantaloupe melon
Strawberries	4079	FOOD00083	Strawberry
Blueberries	3985	FOOD00211	Canada blueberry
Blackberries	4252	FOOD00906	Blackberry
Tap water		N/A	
Coffee, brewed	4433	FOOD00059	Coffee
Afternoon snack			
Tap water		N/A	
Apple, medium	4318	FOOD00105	Apple
Walnuts, NFS	4029	FOOD00608	Walnut
Dinner			
Beef, top sirloin, choice, separable lean, 1/4″ fat, pan fried	42,522	FOOD00495	Cattle (Beef, Veal)
Beans, string, green, raw	4223	FOOD00883	Green bean
Asparagus	4192	FOOD00021	Asparagus
Squash, butternut	4013	FOOD00317	Butternut squash
Oil, olive	8	FOOD00909	Olive oil
Tap water		N/A	
Kiwi fruit, raw	4062	FOOD00004	Kiwi
Evening snack			
Tap water		N/A	
Figs, raw	4197	FOOD00081	Fig
Banana	4036	FOOD00208	Banana
TOTAL	185,937		

## Data Availability

No new data were created or analyzed in this study. Data sharing is not applicable to this article.

## Supplementary Materials

The following supporting information can be downloaded at: https://www.mdpi.com/article/10.3390/metabo14070379/s1, Table S1: Dietary analysis of a whole food diet and an ultra-processed food diet.
