# Supplementary material for: Unraveling the Evolutionary Diet Mismatch and Its Contribution to the Deterioration of Body Composition
Source: Metabolites. 2024 Jul 7;14(7):379. doi: 10.3390/metabo14070379 (PMC11279030; doi:10.3390/metabo14070379)
Supplement: Supplementary file 1 [file metabolites-14-00379-s001.zip › metabolites-3062854-supplementary.pdf]

Supplemental Table S1. Dietary analysis of a whole food diet and an ultraprocessed food diet.

**Nutrient**	**Unit**	**Whole food diet**	**Ultraprocessed food diet**
Energy	kcal	2948.795	3492.075
Total protein	g	174.810	106.643
% energy	%	22.800	12.100
Total carbohydrate	g	192.113	464.482
% energy	%	25.100	52.700
Dietary fiber	g	54.042	23.028
Sugars	g	110.414	162.810
Total fat	g	177.039	137.759
% energy	%	52.100	35.200
Saturated fat	g	33.672	43.317
Monounsaturated fat	g	79.954	30.585
Polyunsaturated fat	g	48.408	17.715
Cholesterol	mg	868.550	112.620
omega-6:omega-3 ratio		8.2:1	16:1
Sodium	mg	1785.052	4623.079
Potassium	mg	8129.252	2379.677
Potassium:sodium ratio		4.5:1	0.5:1
Phosphorus	mg	2283.796	1364.185
Calcium	mg	1197.925	1601.067
Iron	mg	39.002	35.211
Zinc	mg	23.857	9.431
Iodine	mg	9.000	—
Magnesium	mg	943.428	209.895
Copper	mg	4.007	0.762
Manganese	mg	4.605	1.302
Selenium	μg	134.413	73.636
Fluoride	mg	2131.499	897.405
Chromium	mg	0.041	—
Molybdenum	mg	15.445	—
Choline	mg	890.205	23.791
Vitamin A	μg	3543.677	728.800
Vitamin D	μg	3.370	6.294
Vitamin E	mg	28.866	9.483
Vitamin K	mg	2105.943	14.940
Vitamin C	mg	472.104	34.038
Vitamin B_{12}	μg	7.615	8.948
Vitamin B_6	mg	5.151	4.563
Biotin	μg	47.933	—
Pantothenic Acid	mg	11.693	4.331
Niacin	mg	54.021	40.647
Thiamin	mg	2.594	2.771
Riboflavin	mg	4.071	2.795
Folate	μg	1290.215	620.170
